# Filling Teeth

**Published:** 1859-04

**Authors:** Samuel Mallett


### ARTICLE III.

*Filling Teeth.* By SAMUEL MALLETT, D. D. S.

As by an exchange of views and different modes of practice, we arrive at the best way of doing everything relating to dentistry; it would seem clear that the most efficient manner of accomplishing it is for each member of the profession, candidly to detail his own system of practice, not to see what novel thing or finely elaborated theory may be written. We have had a sufficiency for the present, of what has been styled "Fancy Dentistry;" not but that a detail of this may be useful in its place, it is desirable to have it; it has been the means of starting inquiry and thought, and has led the mind to look beyond the beaten track into the fruitful field of experiment and investigation. But what we most want, is an exposition of the daily practice of different individuals, let every man show how *he* operates or manipulates in his ordinary practices; how he does this and that thing.

Astounding performances, or what a man can imagine might be done under certain circumstances, is not what is wanted. If we wished to learn the style of architecture of any country with which we were unacquainted, we should not look for the description of some singular or unique structure, the production of a very imaginative genius of an architect, but entirely wanting in judgment and correct taste. But, on the other hand, should we not seek for the productions of the most approved artists, the actual style of their present edifices. Or, if we wished to know to what extent they had gone in the art of surgery, we should not be the most interested in the examination of the details of a story in which it was stated that a snake had been extracted from the stomach of an individual, but we should first desire to learn to what extent they understood the principles and actually practiced in the most useful and approved as well as difficult operations of surgery.

If all the laboratories and dental chairs could speak, whether in the finest style of English composition or not, we should have a most valuable fund of information for the dental practice.

It is to be feared that many of our most skillful and scientific operators on the natural teeth, as well as manipulators in the laboratory, never reveal to the profession their superior skill, while some of the more superficial, being somewhat accustomed to write, take a higher stand before their brethren, while at the same time they are vastly their inferiors.

What we want, is to hear from the *working* men, the men who blister their fingers, and wear away their finger nails, and raise hard thick pads of skin on the palms of their hands, in making firm rock-like plugs ; the men who actually "earn their bread by the sweat of their brow," in constructing artificial dentures. This may seem a singular exordium to an article on filling teeth, but these thoughts have occurred to me while thinking of the subject, and as I am not "college bred," if I write at all, I must be allowed to write in my own way.

The manner in which I am filling teeth at the present time, may be described as follows : The cavity being suitably prepared, a whole or a part of a sheet of foil, according to the size of the cavity, is folded together and twisted carefully into a rope, which is cut into pieces of various lengths, from one-eighth of an inch to one inch and a quarter long, then with a suitably pointed instrument, one of the longest pieces is taken by one end on the point of the instrument and carried to the place prepared for beginning, (which is often some notch or depression in the cavity,) and firmly pressed against the wall, to which it will usually adhere, if there is any difficulty in causing it to become attached, it should be held there with an instrument or supported by the fingers until a fold can be given it, when no more difficulty will afterward be experienced. The gold can then be very skillfully handled with this pointed instrument, and packed in folds against all the sides of the cavity, each layer being punctured and driven into the preceding one by the sharp instrument point, and so brought home to the wall of the cavity and to every preceding layer, the plug taking the exact form of the cavity, entering into every pit or irregularity of it, and forming when finished, one complete solid piece as if molten. The operation of folding in the foil is continued with a heavy outward pressure until the cavity is full, finishing off with the smaller pieces near the central part of the plug, let the gold go against the wall all around with great force, quite as much as the edge of the cavity will admit, the plug should be packed solidly, especially against all the walls of the cavity. If there must be any part less dense than another, let it be the central portion of the cavity. If this part *alone* is deficient in density the tooth will be saved.

It is best to fill solidly as the operations proceed, so that there shall be no pregnable points to be filled afterwards.

The greatest care should be taken with folding of the foil, and in carrying it to its proper place, in uniform layers of suitable lengths, that the projection of them from the orifice may be just sufficient and not too much folding in the end of each piece of foil, that when the cavity is full, the layers may all be uniform, extending from the base of the cavity to the orifice, as if it were filled with cylinders, but having the advantage over cylinders of better adaptation and greater density, the plug being in one solid mass. If the cavity is very deep, it is best to fill half its depth first and then begin anew. When the cavity is full, the projecting ends are brought together and compressed. Then a heavier instrument is used, flattened and serrated for general and direct pressure upon the whole mass, with a rolling motion; rolling towards the centre of the cavity. This instrument firmly unites the surface of the plug, seals up and completes the unity of the whole.

A plug inserted in this manner, will need scarcely any cutting away, if proceeded with carefully and properly packed, just a sufficiency of foil may be used, and there will be no necessity of touching it with a file or scraper, except for smoothing and polishing; this we consider to be of great importance, as the surface which has received the direct pressures of the serrated instrument, in condensing the plug, is much denser than any surface which may be made, if this should be removed. It is then, with an old file or serrated blade with pumice, by thoroughly rubbing the plug with it, prepared for polishing. Next follows the stone, burnisher, pumice upon a stick, &c., until the highest possible polish is attained.

Too great caution cannot be observed to avoid injuring the edges of the enamel around the cavity. They are in danger of being crushed by the burnisher, and though not enough to cause the fractured part to fall away or even be observed, may, nevertheless, materially injure the value of the operation.

Many very superior plugs are rendered comparatively worthless by the last strokes of the operator. I have destroyed some plugs which had caused the flush of excitement to rush to my cheeks, over an operation brought to a successful issue, which I had considered almost impossible.

The pluggers for this mode of filling, are very simple and easily made, being nearly square and almost sharp pointed, with a slight temper, curved about one-eighth of an inch from the point. They are bent after being tempered, and readily altered to suit the peculiarity of any cavity. For approximal cavities near the gum, an instrument shaped like the blade of a gum lancet, with serrated sides, should be used to draw the projecting foil from the gum and condense it there.

To Dr. Benjamin Lord, of New York, I am indebted for important suggestions regarding this manner of filling teeth, and I have seen as good plugs by him, which were made in this way, as have ever come under my observation, by any other mode or form of gold. And, though I may say with Dr. Taylor, that "If any man is making good plugs by any particular mode of practice, he would not advise him to change." I may safely say, that if all members of the profession would adopt this mode of filling, more teeth would be saved than there are.

After experimenting in various ways, with the modern preparations of gold, and new modes of working them, I have settled down on this, and now fill fifty teeth in this way to any other. There is an advantage in filling all cavities in one way, or as nearly so as possible—a person cannot do as well if he is changing about from one mode to another.

If I have a cavity which it is almost impossible to fill, on account of the excessive flow of saliva, I use cylinders, but consider that I do not make as perfect plugs as by the mode I have described; the union of the cylinders cannot be as complete with the walls of the cavity, as with each other.

If I wish to build up the crown of a tooth, I use crystal gold or crystalline gold foil, but this I never do except to assist in mastication. Here, among the Yankees, we do not consider that it helps the appearance of a front tooth to restore the lost portion with gold, and I will say frankly, we do not generally find people willing to make so great a sacrifice of time and money, as is required to build out teeth, unless some useful purpose is to be secured.

It is my custom to destroy exposed nerves, extirpate them, and fill to the fang. My success has been very flattering and satisfactory in this practice. Two remarkable cases I will name, which occurred at the same time, and were so nearly alike, both first right inferior molars, that to describe one would be to describe the other. The Rev. Dr. —— called on me late in the evening to go and see his wife, who for four days had been suffering most intensely with a tooth which had been quackishly filled with amalgam on to the bare pulp. I removed the amalgam and plug, and applied arsenic and kreosote, and directed the patient to call in a day or two and have the tooth plugged, but she had immediate relief, and did not have the tooth filled in several weeks. I then cleansed the tooth to the fang and filled it as I did also the other, and they have both given no trouble whatever, notwithstanding several weeks elapsed between the time of destroying the pulp and filling the teeth, and that they had given great pain previous to the operation. Two years have now passed since these were filled.

I mention these because they were cases, which at the time of the operation, were very doubtful as to their result, and, in my opinion, speak much for fang filling.

